# Single-Nucleotide Polymorphisms in Capecitabine Bioactivation Genes and Their Contribution to Breast Cancer Therapy

**DOI:** 10.3390/pharmaceutics18060633

**Published:** 2026-05-22

**Authors:** Andrea Fernández, Yasmín Cura-Cuevas, Susana Rojo-Tolosa, José María Gálvez-Navas, Encarnación González-Flores, Cristina Pérez-Ramírez, Alberto Jiménez-Morales

**Affiliations:** 1 Pharmacy Service, Pharmacogenetics Unit, Hospital Universitario Virgen de las Nieves, 18014 Granada, Spain; susanarojotolosa@gmail.com (S.R.-T.); alberto.jimenez.morales.sspa@juntadeandalucia.es (A.J.-M.); 2 Instituto de Investigación Biosanitaria ibs., 18012 Granada, Spain; yasmin.cura.easp@juntadeandalucia.es (Y.C.-C.); jmaria.galvez.easp@juntadeandalucia.es (J.M.G.-N.); encarnacion.gonzalez.flores.sspa@juntadeandalucia.es (E.G.-F.); 3 Cancer Registry of Granada, Andalusian School of Public Health, Cuesta del Observatorio 4, Campus Universitario de Cartuja, 18011 Granada, Spain; 4 Center of Biomedical Research, Department of Biochemistry and Molecular Biology II, Institute of Nutrition and Food Technology “José Mataix”, University of Granada, 18071 Granada, Spain; cperezramirez87@ugr.es; 5 Pneumology Department, Hospital Universitario Virgen de las Nieves, Avda. de las Fuerzas Armadas 2, 18014 Granada, Spain; 6 Centro de Investigación Biomédica en Red de Epidemiología y Salud Pública (CIBERESP), 18016 Granada, Spain; 7 Medical Oncology, Hospital Universitario Virgen de las Nieves, 18014 Granada, Spain

**Keywords:** breast cancer, capecitabine, bioactivation, single-nucleotide polymorphisms, survival

## Abstract

**Background/Objectives:** Breast cancer (BC) is a highly prevalent neoplasm worldwide. Despite the wide range of therapeutic options currently available, it remains the leading cause of cancer-related mortality among women. Capecitabine, a prodrug of 5-fluorouracil (5-FU), is widely used in the treatment of advanced BC. However, despite its efficacy, capecitabine exhibits considerable interindividual variability in therapeutic response. This study aimed to evaluate the effect of single-nucleotide polymorphisms (SNPs) in genes involved in capecitabine bioactivation on progression-free survival (PFS) in patients with BC. **Methods:** An ambispective cohort study was conducted. Four relevant SNPs in the *CES1*, *CDA*, and *TYMP* genes were analyzed in 85 Caucasian patients with BC using real-time polymerase chain reaction (PCR) with TaqMan^®^ probes. **Results:** A significant association was observed between shorter PFS and the GA genotype of the *CES1 rs71647871* SNP (*p =* 0.010; HR = 7.46; 95% CI = 1.24–122.52), as well as with the TT genotype of the *CDA rs602950* SNP (*p =* 0.009; HR = 3.50; 95% CI = 1.36–9.03). **Conclusions:** These findings suggest that *CES1 rs71647871* and CDA *rs602950* may serve as predictive biomarkers of capecitabine effectiveness in patients with BC. Further studies involving larger cohorts are needed to validate these findings and generate additional evidence to support their potential implementation in clinical practice.

## 1. Introduction

Breast cancer (BC) is a major global health concern. It is the second most frequently diagnosed neoplasm after colorectal cancer and the leading cause of cancer-related death among women [1]. According to its histological subtype, BC can be classified as ductal carcinoma in situ (DCIS), invasive ductal carcinoma (IDC), invasive lobular carcinoma (ILC), invasive carcinoma (IC), or mucinous carcinoma (MC), among others [2]. Therapeutic strategies are determined by tumor characteristics and hormone receptor status, resulting in a wide range of treatment options, including local interventions (surgery or radiotherapy) and systemic approaches (chemotherapy, hormone therapy, targeted therapies, or immunotherapy) [3,4].

Capecitabine, an oral prodrug of 5-fluorouracil (5-FU), is widely used in the treatment of advanced and metastatic BC, either as monotherapy or in combination with other antineoplastic agents [5]. Compared with 5-FU, capecitabine offers the convenience of oral administration and is generally associated with a more favorable safety profile [6]. Although capecitabine has demonstrated significant clinical efficacy across several tumor types [7], considerable interpatient variability in therapeutic response and toxicity has been reported [5]. This variability may be attributed to numerous demographic and clinical factors, such as age, diet, nulliparity, and family history of cancer, but it may also have a genetic basis [8,9]. Genetic polymorphisms, particularly single-nucleotide polymorphisms (SNPs), may influence the expression or activity of metabolizing and transport proteins, as well as transcription factors binding sites, thereby affecting the expression of genes and proteins involved in detoxification and ultimately altering treatment outcomes [7,10].

Currently, there are no predictive biomarkers with established clinical utility for assessing capecitabine effectiveness. Four genetic variants in the *DPYD* gene: *rs3918290* (DPYD*2A), *rs55886062* (DPYD*13), *rs67376798*, and *rs75017182*/*rs56038477* (HapB3), have demonstrated sufficient clinical evidence linking them to capecitabine-induced toxicity [11]. *DPYD* encodes dihydropyrimidine dehydrogenase (DPD), the rate-limiting enzyme in fluoropyrimidine catabolism. However, these variants account for only a small proportion of toxicity cases, suggesting that additional genetic factors may contribute to the substantial interindividual variability in treatment outcomes [12].

**Figure 1:**
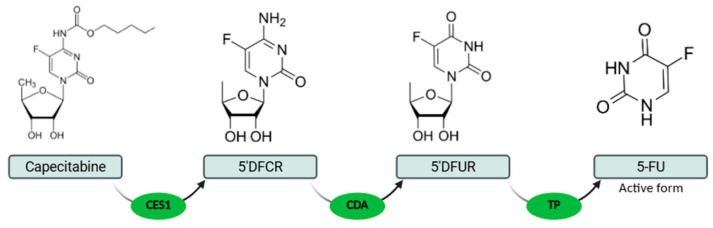
Capecitabine bioactivation pathway. Created with Biorender.

One of the capecitabine pharmacokinetic pathways currently receiving considerable attention is its bioactivation. This process involves three sequential enzymatic steps (Figure 1). In the liver, capecitabine is hydrolyzed by carboxylesterase 1 (CES1) to form 5′-deoxy-5-fluorocytidine (5′-DFCR). Cytidine deaminase (CDA) subsequently converts 5′-DFCR to 5′-deoxy-5-fluorouridine (5′-DFUR). Finally, thymidine phosphorylase (TP), which is more highly expressed in tumor tissues than in normal tissues, catalyzes the conversion of 5′-DFUR to the active metabolite 5-FU [13,14]. Given the critical role of these enzymes in the bioavailability of the active compound, genetic variations affecting their activity could directly influence treatment outcomes. However, pharmacogenetic associations related to capecitabine effectiveness have shown inconsistent results across studies, potentially reflecting heterogeneity in cancer type, treatment regimens, study design, and ethnic background of patient populations [10,15,16,17].

The limited evidence regarding the association between genetic variants involved in capecitabine bioactivation and treatment effectiveness in BC highlights the need for further research. Therefore, this study aimed to evaluate the potential effect of SNPs in *CES1*, *CDA*, and *TYMP* on treatment effectiveness in Caucasian patients with BC from southern Spain.

## 2. Materials and Methods

### 2.1. Study Design and Ethical Considerations

This research was designed as an observational, ambispective study and was conducted in accordance with the principles of the Declaration of Helsinki. DNA samples were obtained with the approval of the Biomedical Research Ethics Committee of Granada (identification code 0632-M2-20, 2020) and were organized alphanumerically to ensure confidentiality. All patients provided written informed consent for the donation of these samples to the Biobank of the Andalusian Public Health System.

### 2.2. Study Population

DNA samples from patients with BC who had previously undergone *DPYD* genotyping at Hospital Universitario Virgen de las Nieves (HUVN) in Granada were requested from the Biobank of the Andalusian Public Health System and stored at −40 °C in the Pharmacogenetics Unit of HUVN (Granada, Spain). Samples were selected according to the following inclusion criteria: (1) diagnosis of BC, (2) age ≥ 18 years, and (3) treatment with capecitabine-based therapy. Patients with inaccessible medical records or who had received capecitabine <12 weeks were excluded. A total of 156 patients with BC underwent *DPYD* genotyping during the recruitment period (2021–2024). Of these, 111 received capecitabine therapy and were initially included in the study. Subsequently, 26 patients were excluded due to insufficient treatment duration or inaccessible medical records, resulting in a final cohort of 85 patients who met the selection criteria (Figure S1).

#### Dosage

Capecitabine was administered at daily doses ranging from 1500 to 4300 mg for 14 consecutive days in 3-week cycles. Patients received capecitabine either as monotherapy or in combination with other antineoplastic agents. Treatment continued until completion of the planned cycles, disease progression, development of severe toxicity, or death. In cases of severe toxicity, capecitabine dose reductions were performed when clinically indicated to avoid treatment discontinuation.

### 2.3. Variables

#### 2.3.1. Outcome Variable

Progression-free survival (PFS), defined as the time from initiation of capecitabine-based therapy to disease progression or death, was obtained from medical records.

#### 2.3.2. Sociodemographic and Clinical Variables

Sociodemographic and clinical data were collected from medical records documented by the HUVN Medical Oncology Department during routine follow-up visits using the Diraya Clinical Station of the Andalusian Health System. These variables included sex; age at BC diagnosis; family history of cancer and BC (yes/no); smoking status (smoker/non-smoker/former smoker): alcohol consumption (drinker/non-drinker/former drinker): molecular subtype of BC (Luminal A, Luminal B, HER2+, and Basal); tumor histopathology (DCIS, IDC, ILC, IC, and MC); capecitabine treatment setting (non-adjuvant/adjuvant); treatment line (1º/2º/≥3º); and dose reduction due to toxicity (yes/no). Patients were classified according to receipt of adjuvant therapy. Cases not receiving adjuvant treatment were grouped as non-adjuvant because the dataset did not allow reliable distinction between metastatic and neoadjuvant settings.

#### 2.3.3. Genetic Variables

DNA quality and purity were assessed by measuring the A260/A280 and A260/A230 ratios using a NanoDrop 2000 UV spectrophotometer (ThermoFisher Scientific^®^, Waltham, MA, USA). Four SNPs in the *CES1* (*rs71647871*), *CDA* (*rs602950*, *rs1048977*), and *TYMP* (*rs11479*) genes were selected based on existing literature regarding their impact on capecitabine therapy outcomes and their minor allele frequency (MAF) in the Iberian population (>1%) [18,19,20,21] (Table 1).

Genotyping was performed using TaqMan™ probes and real-time PCR on a QuantStudio^®^ 3 thermocycler (Thermo Fisher Scientific, Waltham, MA, USA) following the manufacturer’s instructions. Samples were analyzed in multiple batches using 96-well plates in the Pharmacogenetics Unit at HUVN. In addition, 10% of the samples were analyzed in duplicate. Genotyping quality was satisfactory for all SNPs, with concordance rates > 98%.

### 2.4. Statistical Analysis

The Kolmogorov–Smirnov test was used to assess data normality. Qualitative variables were expressed as frequencies and percentages, whereas quantitative variables were expressed as mean ± standard deviation (SD) or median [p25–p75], as appropriate. Genotype frequencies were tested for Hardy–Weinberg equilibrium (HWE) using PLINK v1.9, excluding SNPs with *p*-value < 0.050. Linkage disequilibrium (LD) and the corresponding plots were analyzed using PLINK v1.9 and Haploview v.4 software [22,23].

Associations between PFS, clinical and sociodemographic variables, and SNPs were assessed through bivariate analysis using Kaplan–Meier curves, the log-rank test, and Cox proportional hazards regression models. Variables showing statistical significance in the bivariate analysis were subsequently included in multivariate Cox regression models. The genetic models evaluated included dominant, recessive, and genotypic models. Statistical significance was established at *p* ≤ 0.05. Multiple testing correction was performed using the false discovery rate (FDR) method to control for type I error, and FDR-adjusted *p*-values < 0.05 were considered statistically significant.

All analyses were conducted using PLINK v1.9 and R v.4.2.2 software [22,24].

## 3. Results

### 3.1. Sociodemographic and Clinical Characteristics

The study included a total of 85 patients diagnosed with BC. The sociodemographic and clinical characteristics of the cohort are presented in Table 2. All patients were female (100%). The mean age at diagnosis was 50.22 ± 11.93 years. Among participants, 69.41% (59/85) had a family history of cancer, 37.64% (32/85) had a family history of BC, and 72.83% (59/81) presented with IDC histopathology. The median daily capecitabine dose was 3300 [3000–3500] mg. Additionally, 15.29% (13/81) of patients received capecitabine in the adjuvant setting, whereas 83.95% (68/81) received it in a non-adjuvant setting. Furthermore, 68.23% (58/85) of patients received capecitabine as a third-line or later treatment, and 45.88% (39/85) required dose reductions due to severe toxicity. The median follow-up time was 29.13 [8.57–101.77] months.

### 3.2. Genotype Distribution

None of the analyzed SNPs were found to be in LD (Figure S2). The MAF for all SNPs analyzed was >1% (Table S1). All SNPs were in HWE (*p* > 0.05) (Table S2).

### 3.3. Influence of Sociodemographic and Clinical Characteristics on Progression-Free Survival

Patients who received capecitabine in the non-adjuvant setting exhibited shorter PFS (*p =* 0.009; HR = 4.41; 95%CI = 1.07–18.17, for adjuvant vs. non-adjuvant) (Table S3; Figure S3). Similarly, patients treated in the third-line or later setting showed shorter PFS (*p =* 0.020; HR = 4.82; 95%CI = 1.17–19.93, for first-line vs. ≥third-line) (Table S3; Figure S4). No other significant associations were identified between PFS and the remaining sociodemographic or clinical variables (Table S3).

### 3.4. Influence of SNPs Involved in Capecitabine Bioactivation on Progression-Free Survival

**Figure 2:**
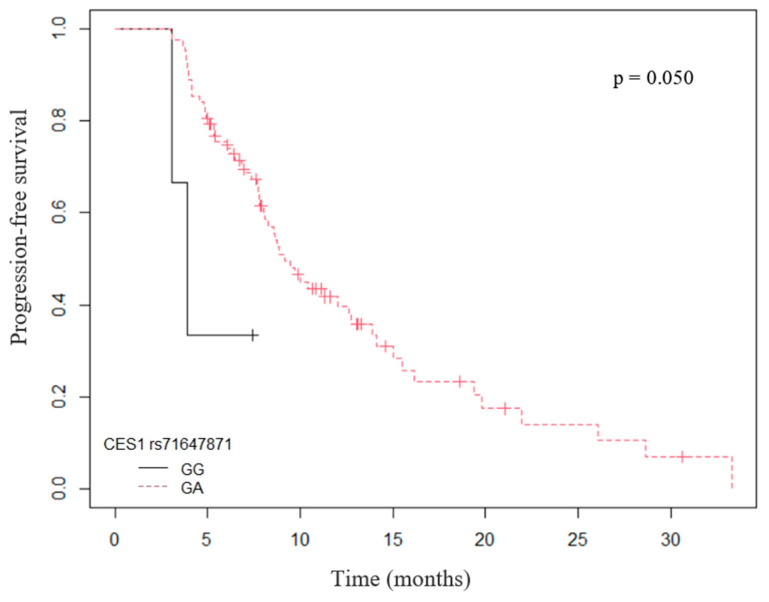
Kaplan–Meier curves for PFS according to the GA genotype of the SNP *CES1 rs71647871* (GG vs. GA).

**Figure 3:**
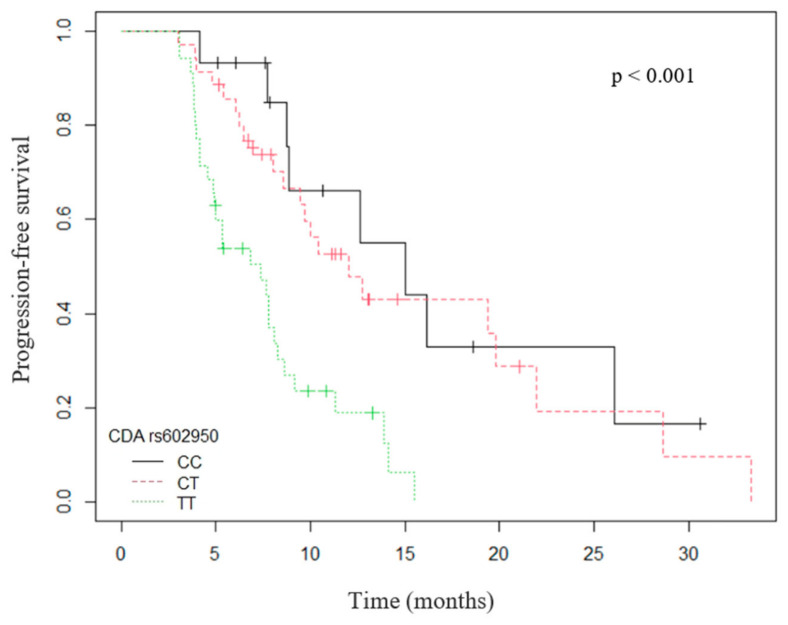
Kaplan–Meier curves for PFS according to the TT genotype of the SNP *CDA rs602950* (CC vs. TT).

A significant association was observed between shorter PFS and the GA genotype of the *CES1 rs71647871* SNP (*p =* 0.050; HR = 3.90; 95%CI = 0.92–16.54 for GG vs. GA and A vs. GG) (Table S3; Figure S5). Similarly, the TT genotype of the *CDA rs602950* SNP was associated with shorter PFS (*p* < 0.001; HR = 3.93; 95%CI = 1.72–8.96 for CC vs. TT, and *p* < 0.001; HR = 3.25; 95%CI = 1.86–5.69 for C vs. TT) (Table S3; Figure S6). Kaplan–Meier curves in Figure 2 and Figure 3 illustrate the associations between PFS and the *CES1 rs71647871* and *CDA rs602950* SNPs, respectively. No additional associations were identified between PFS and the remaining analyzed SNPs (Table S3).

Multivariate analysis confirmed significant associations between shorter PFS and the GA genotype of *CES1 rs71647871* (*p =* 0.010; HR = 7.46; 95%CI = 1.24–122.52, for GG vs. GA), as well as with the TT genotype of *CDA rs602950* (*p =* 0.009; HR = 3.50; 95%CI = 1.36–9.03, for CC vs. TT) (Table 3). These associations remained significant after FDR correction. The wide 95% confidence interval observed for *CES1 rs71647871* likely reflects the limited sample size and the low frequency of the variant in the study cohort. Therefore, this estimate should be interpreted with caution.

## 4. Discussion

Currently, there are no predictive biomarkers for capecitabine in patients with BC, underscoring the need to expand research efforts toward other genes involved in capecitabine pharmacokinetics. The bioactivation pathway of capecitabine involves multiple genes that may influence therapeutic outcomes in BC. In the present study, a significant association was observed between shorter PFS and both the GA genotype of *CES1 rs71647871* and the TT genotype of *CDA rs602950* in patients with BC treated with capecitabine.

Carboxylesterases (CES1 and CES2) belong to the α/β-hydrolase protein family and are encoded by the *CES* gene family located on chromosome 16. CES1 is the most abundantly expressed hydrolytic enzyme in human liver tissue and participates in the metabolism of numerous drugs [25,26]. CES1 plays a crucial role in capecitabine bioactivation by hydrolyzing it to 5′-DFCR [27]. Among the most extensively studied SNPs in the *CES1* gene related to capecitabine toxicity is *rs71647871* (c.428G>A), which results in a Gly143Glu amino acid substitution and subsequent loss of enzymatic activity [28]. In this study, the GA genotype of *CES1 rs71647871* was associated with shorter PFS (Table 3). To date, no studies have identified an association between *CES1 rs71647871* and capecitabine effectiveness. However, *CES1 rs71647871* SNP has previously been investigated regarding its impact on capecitabine-related toxicity. Cura et al. (Caucasian population; Spain; n = 161) reported that carriers of the *CES1 rs71647871*-A allele had a higher likelihood of experiencing overall toxicity (*p =* 0.044) and severe HFS (*p =* 0.030) among patients with colorectal cancer treated with capecitabine [29]. In contrast, Hamzic et al. (mixed population; n = 144) did not identify significant associations between *CES1 rs71647871* and capecitabine-related toxicity in cancer patients [30]. The loss of enzymatic function caused by this variant could plausibly affect therapeutic effectiveness. Reduced capecitabine hydrolysis may lead to lower formation of the active metabolite, potentially resulting in decreased therapeutic efficacy and subsequent disease progression.

CDA is an enzyme that catalyzes the hydrolytic deamination of cytidine to uridine and deoxycytidine to deoxyuridine. It belongs to the family of zinc-dependent cytidine and deoxycytidylate deaminases and is encoded by the *CDA* gene located on chromosome 1 [31,32]. CDA contributes to pyrimidine catabolism, and alterations in its enzymatic activity may directly affect cancer therapy outcomes. CDA overexpression has been associated with chemotherapy resistance and reduced therapeutic effectiveness, whereas CDA deficiency has been linked to severe early toxicity to agents such as gemcitabine [32,33]. *CDA rs602950* (c.92C>T), located in the promoter region, is among the most extensively studied SNPs in Caucasian populations. This variant affects the 5′UTR region and has been associated with reduced CDA enzymatic activity [34]. In the present study, the TT genotype of *CDA rs602950* was associated with shorter PFS in BC patients treated with capecitabine (Table 3). In contrast, Liu et al. (Asian population, n = 322; 2019) reported a significant association between the CC genotype of *CDA rs602950* and shorter PFS (*p =* 0.002) in patients with gastric or colorectal cancer receiving capecitabine-based neoadjuvant therapy. Notably, that study also identified strong correlations between *CDA rs2072671* and *rs532545* (tag SNPs for *rs602950*) and PFS, liver toxicity, and hematologic toxicities [20]. Similarly, Martin et al. (Caucasian population, Spain; n = 195) found a significant association between the *CDA rs602950*-C allele and reduced PFS (*p =* 0.038) in patients with metastatic BC treated with capecitabine [35]. In addition, *CDA rs602950* has been extensively investigated regarding its influence on capecitabine. Several studies have associated the presence of the *CDA rs602950*-T allele with severe diarrhea in cancer patients receiving capecitabine-based therapy [30,36]. Given the role of CDA in capecitabine metabolism, the association observed in the present study is biologically plausible and suggests a potential role for this variant as a predictive biomarker of treatment response. However, the absence of a comparator group precludes fully distinguishing this effect from a possible prognostic component, which should be addressed in future studies. Some studies have demonstrated that the clinical impact of *CDA* polymorphisms is not uniform across populations due to variability in allele frequencies among different ethnic groups. In addition, differences in *CDA* expression across tumor types may contribute to the heterogeneous toxicity and therapeutic outcomes associated with identical *CDA* variants depending on ethnic background and cancer type [31,37,38]. Furthermore, because *CDA rs602950* is located within the promoter region, its functional impact on CDA transcription may be highly context-dependent. This polymorphism may alter transcription factor binding and gene expression, which can be influenced by epigenetic regulation, cellular signaling pathways, and tumor microenvironment conditions. These mechanisms may further contribute to the heterogeneous clinical effects observed across different cancer types and patient populations [31,36]. Therefore, the discrepancies between present findings may be partly explained by differences in treatment regimens, ethnic background, and cancer type, underscoring the need for further studies to clarify the relationship between *CDA rs602950* and capecitabine treatment outcomes.

The impact of SNPs on CES1 and CDA enzymatic activity may directly influence capecitabine treatment outcomes. However, evidence regarding the effects of *CES1 rs71647871* and *CDA rs602950* in patients with BC remains limited. Further studies are needed to validate these variants as predictive biomarkers of capecitabine effectiveness in BC patients.

Several limitations of this study should be acknowledged. First, the relatively small sample size may have limited the ability to detect additional associations between treatment effectiveness and other SNPs involved in capecitabine bioactivation. Moreover, because no previous studies have investigated the role of *CES1 rs71647871* in capecitabine effectiveness in BC or other cancer types, the findings reported here require further validation. Although the observed association is novel and statistically significant, the 95% CI (1.24–122.52), highlights the need for validation in larger cohorts. In addition, the study population consisted exclusively of Caucasian patients with BC, which may limit the generalizability of the findings to other ethnic groups or cancer types. Furthermore, since the sample size was not calculated but rather consisted of all subjects who could be recruited during the established inclusion period, the study’s statistical power may have been limited, increasing the risk of Type II errors and reducing the ability to detect significant associations and to generalize the results to other populations. The findings should therefore be interpreted with caution and considered primarily as a basis for generating hypotheses for future studies with a prospectively calculated sample size. Finally, the inability to distinguish between metastatic and neoadjuvant settings within the non-adjuvant group represents an additional limitation and may have introduced clinical heterogeneity.

Despite these limitations, this study represents a novel contribution, as no previous research has investigated the potential role of these specific SNPs in capecitabine effectiveness among patients with BC. Even with a relatively limited cohort, several statistically significant associations were identified, providing a foundation for future investigations. Although the findings support the potential role of these SNPs as predictive biomarkers of capecitabine effectiveness, further research in larger and more diverse cohorts is necessary to confirm their clinical utility.

## 5. Conclusions

In patients with BC, the GA genotype of *CES1 rs71647871* and the TT genotype of *CDA rs602950* were associated with reduced effectiveness of capecitabine therapy. These findings support the hypothesis that genetic variants in genes involved in capecitabine bioactivation may serve as predictive biomarkers of therapeutic response. The limited available evidence and the inconsistencies among existing findings underscore the need for continued research in this field. Studies involving larger and more diverse cohorts could not only validate the findings of the present study but also provide additional evidence regarding the role of pharmacogenetic variants in capecitabine effectiveness in BC.

## Figures and Tables

**Table 1:** Characteristics of the selected SNPs.

Gene	SNP	Chr	Allele Change	SNP Type	TaqMan^®^ Assay ID
*CDA*	*rs602950*	1	A>G	2KB upstream variant	-
*CDA*	*rs1048977*	1	C>T	Synonymous variant	C___7477307_30
*CES1*	*rs71647871*	16	G>A	Missense variant	-
*TYMP*	*rs11479*	22	C>T	Missense variant	C__11946264_20

Chr: chromosome; SNP: single-nucleotide polymorphism; -: custom TaqMan^®^ Probe.; A: adenine; C: cytosine; G: guanine; T: thymine.

**Table 2:** Sociodemographic and clinical characteristics of the 85 BC patients included in the study.

Characteristic		N	%
Sex	Female	85	100
Family history of cancer	Yes	59	69.41
	No	26	30.59
Family history of BC	Yes	32	37.65
	No	53	62.35
Age at BC diagnosis (years)		50.22 ± 11.93	
Smoking status	Smoker	14	16.47
	Non-smoker	60	70.59
	Former smoker	11	12.94
Alcohol consumption	Drinker	0	0
	Non-drinker	84	98.82
	Former drinker	1	1.18
Molecular subtype	Luminal A	15	17.65
	Luminal B	35	41.18
	HER2+	2	2.35
	Basal	24	28.23
	Missing	9	10.59
Histopathology	DCIS	4	4.70
	IDC	59	69.41
	ILC	12	14.12
	IC	5	5.88
	MC	1	1.18
	Missing	4	4.71
Treatment setting	Adjuvant	13	15.29
	Non-adjuvant	68	80.00
	Missing	4	4.71
Treatment line	1º	13	15.29
	2º	14	16.47
	≥3º	58	68.24
Capecitabine dose reduction	Yes	39	45.88
	No	46	54.12
Capecitabine daily dose (mg)		3300 [3000–3500]	

N (%), mean ± SD, or median [IQR]. BC: breast cancer; DCIS: ductal carcinoma in situ; IC: inflammatory carcinoma; IDC: invasive ductal carcinoma; ILC: invasive lobular carcinoma; MC: mixed mucinous carcinoma.

**Table 3:** Multivariate Cox regression analysis of PFS associations with sociodemographic, clinical, and SNPs in capecitabine bioactivation pathway.

	Progression-Free Survival		
	HR (95%CI)	*p*-Value	p-BH *
*CES1 rs71647871* (GA)	7.46 (1.60–34.73)	0.010	0.050
*CDA rs602950* (TT)	3.50 (1.36–9.03)	0.009	0.025
Model *p*-value < 0.001			

HR: Hazard ratio. 95%CI: 95% confidence interval. Model adjusted by treatment setting and line * Benjamini–Hochberg adjusted *p*-value.

## Data Availability

The data presented in this study are available on request from the corresponding author due to privacy and ethical restrictions.

## Supplementary Materials

The following supporting information can be downloaded at: https://www.mdpi.com/article/10.3390/pharmaceutics18060633/s1, Figure S1: Patient flow diagram; Figure S2: Linkage disequilibrium of selected SNPs. Color scheme: white: D’ < 1, LOD < 2; Table S1: Minor allele frequency of the selected SNPs; Table S2: Hardy–Weinberg Equilibrium Analysis of the selected SNPs; Table S3: Association of sociodemographic and clinical characteristics with PFS; Figure S3: Kaplan–Meier survival curves for PFS according to capecitabine treatment setting (adjuvant vs. neoadjuvant); Figure S4: Kaplan–Meier survival curves for PFS according to capecitabine treatment line (first-line vs. ≥third-line); Figure S5: Kaplan–Meier curves for PFS according to the A allele of the *CES1 rs71647871* SNP (GG vs. A); Figure S6: Kaplan–Meier curves for PFS according to the C allele of the *CDA rs602950* SNP (TT vs. C).

## Abbreviations

5′dFCR: 5′-deoxy-5-fluorocytidine; 5′dFUR: 5′-deoxy-5-fluorouridine; 5-FU: 5-fluorouracil; 95%CIs: 95% confidence intervals; BC: breast cancer; CES1: carboxylesterase 1 enzyme; *CES1*: carboxylesterase 1 gene; CES2: carboxylesterase 2 enzyme; CES2: carboxylesterase 2 gene; CDA: cytidine deaminase enzyme; *CDA*: cytidine deaminase gene; DCIS: ductal carcinoma in situ; DPD: dihydropyrimidine dehydrogenase enzyme; *DPYD*: dihydropyrimidine dehydrogenase gene; FDR: false discovery rate; HapB3: haplotype B3; HR: hazard ratio; HWE: Hardy–Weinberg equilibrium; IBS: Iberian population in Spain; IC: inflammatory carcinoma; IDC: invasive ductal carcinoma; ILC: invasive lobular carcinoma; LD: linkage disequilibrium; MAF: minor allele frequency; MC: mixed mucinous carcinoma; PCR: polymerase chain reaction; SNP: single-nucleotide polymorphism; TP: thymidine phosphorylase enzyme; *TYMP*: thymidine phosphorylase gene.

## References

[1] García A.E., Pérez P. (2025). Cáncer de Mama Keywords. Rev. La Educ. Super..

[2] Tan P.H., Ellis I., Allison K., Brogi E., Fox S.B., Lakhani S., Lazar A.J., Morris E.A., Sahin A., Salgado R. (2020). The 2019 World Health Organization Classification of Tumours of the Breast. Histopathology.

[3] Xiong X., Zheng L.W., Ding Y., Chen Y.F., Cai Y.W., Wang L.P., Huang L., Liu C.C., Shao Z.M., Yu K. (2025). Da Breast Cancer: Pathogenesis and Treatments. Signal Transduct. Target. Ther..

[4] Daniyal A., Santoso I., Gunawan N.H.P., Barliana M.I., Abdulah R. (2021). Genetic Influences in Breast Cancer Drug Resistance. Breast Cancer Targets Ther..

[5] Dean L., Kane M. (2012). Capecitabine Therapy and DPYD Genotype. Medical Genetics Summaries.

[6] Alzahrani S.M., Al Doghaither H.A., Al-Ghafari A.B., Pushparaj P.N. (2023). 5-Fluorouracil and Capecitabine Therapies for the Treatment of Colorectal Cancer (Review). Oncol. Rep..

[7] Vanave A.M. (2023). Capecitabine: A Promising Anticancer Drug. J. Pharma Insights Res..

[8] García-Sancha N., Corchado-Cobos R., Pérez-Losada J. (2025). Understanding Susceptibility to Breast Cancer: From Risk Factors to Prevention Strategies. Int. J. Mol. Sci..

[9] Lai E., Persano M., Dubois M., Spanu D., Donisi C., Pozzari M., Deias G., Saba G., Migliari M., Liscia N. (2022). Drug-Related Toxicity in Breast Cancer Patients: A New Path towards Tailored Treatment?—A Narrative Review. Precis. Cancer Med..

[10] Lam S.W., Guchelaar H.J., Boven E. (2016). The Role of Pharmacogenetics in Capecitabine Efficacy and Toxicity. Cancer Treat. Rev..

[11] Amstutz U., Henricks L.M., Offer S.M., Barbarino J., Schellens J.H.M., Swen J.J., Klein T.E., McLeod H.L., Caudle K.E., Diasio R.B. (2018). Clinical Pharmacogenetics Implementation Consortium (CPIC) Guideline for Dihydropyrimidine Dehydrogenase Genotype and Fluoropyrimidine Dosing: 2017 Update. Clin. Pharmacol. Ther..

[12] De Mattia E., Milan N., Assaraf Y.G., Toffoli G., Cecchin E. (2024). Clinical Implementation of Rare and Novel DPYD Variants for Personalizing Fluoropyrimidine Treatment: Challenges and Opportunities. Int. J. Biol. Sci..

[13] Alqahtani S., Alzaidi R., Alsultan A., Asiri A., Asiri Y., Alsaleh K. (2022). Clinical Pharmacokinetics of Capecitabine and Its Metabolites in Colorectal Cancer Patients. Saudi Pharm. J..

[14] Bai Y., Chen H., Gu L., Shi B., Wang Z., Duanmu Y., Hu Y., Wang Y., Zhang C., Su Z. (2025). Identification of Metabolites Associated with Capecitabine-induced Hand-foot Syndrome Using Untargeted Metabolomics in Patients with Cancer. Mol. Med. Rep..

[15] Chan T.H., Zhang J.E., Pirmohamed M. (2024). DPYD Genetic Polymorphisms in Non-European Patients with Severe Fluoropyrimidine-Related Toxicity: A Systematic Review. Br. J. Cancer.

[16] Sánchez-Bayona R., Catalán C., Cobos M.A., Bergamino M. (2025). Pharmacogenomics in Solid Tumors: A Comprehensive Review of Genetic Variability and Its Clinical Implications. Cancers.

[17] Zhou Y., Lauschke V.M. (2022). Population Pharmacogenomics: An Update on Ethnogeographic Differences and Opportunities for Precision Public Health. Hum. Genet..

[18] De Mattia E., Roncato R., Fratte C.D., Ecca F., Toffoli G., Cecchin E. (2019). The Use of Pharmacogenetics to Increase the Safety of Colorectal Cancer Patients Treated with Fluoropyrimidines. Cancer Drug Resist..

[19] Jia X., Zhang T., Sun J., Lin H., Bai T., Qiao Y., Li Y., Li G., Li G., Peng X. (2023). Rs11479 in Thymidine Phosphorylase Associated with Prognosis of Patients with Colorectal Cancer Who Received Capecitabine-Based Adjuvant Chemotherapy. Pharmgenomics Pers. Med..

[20] Liu D., Li X., Li X., Zhang M., Zhang J., Hou D., Tong Z., Dong M. (2019). CDA and MTHFR Polymorphisms Are Associated with Clinical Outcomes in Gastroenteric Cancer Patients Treated with Capecitabine-Based Chemotherapy. Cancer Chemother. Pharmacol..

[21] Liu D., Li X., Li X., Wang H., Dong M. (2021). Carboxylesterase 1 Polymorphisms Are Associated with Clinical Outcomes in Gastroenteric Cancer Patients Treated with Capecitabine. Cancer Chemother. Pharmacol..

[22] Purcell S., Neale B., Todd-Brown K., Thomas L., Ferreira M.A.R., Bender D., Maller J., Sklar P., De Bakker P.I.W., Daly M.J. (2007). PLINK: A Tool Set for Whole-Genome Association and Population-Based Linkage Analyses. Am. J. Hum. Genet..

[23] Barrett J.C., Fry B., Maller J., Daly M.J. (2005). Haploview: Analysis and Visualization of LD and Haplotype Maps. Bioinformatics.

[24] Lafaye de Micheaux P., Drouilhet R., Liquet B. (2013). The R Software: Fundamentals of Programming and Statistical Analysis.

[25] Wang D., Zou L., Jin Q., Hou J., Ge G., Yang L. (2018). Human Carboxylesterases: A Comprehensive Review. Acta Pharm. Sin. B.

[26] Wang X., Shi J., Zhu H.J. (2019). Functional Study of Carboxylesterase 1 Protein Isoforms. Proteomics.

[27] Stoeva S., Conev N., Marinov P. (2020). The Role of Carboxylesterase Enzymes in Capecitabine Therapy. Scr. Sci. Pharm..

[28] Ikonnikova A., Rodina T., Dmitriev A., Melnikov E., Kazakov R., Nasedkina T. (2022). The Influence of the CES1 Genotype on the Pharmacokinetics of Enalapril in Patients with Arterial Hypertension. J. Pers. Med..

[29] Cura Y., Sánchez-Martín A., Márquez-Pete N., González-Flores E., Martínez-Martínez F., Pérez-Ramírez C., Jiménez-Morales A. (2023). Association of Single-Nucleotide Polymorphisms in Capecitabine Bioactivation Pathway with Adjuvant Therapy Safety in Colorectal Cancer Patients. Pharmaceutics.

[30] Hamzic S., Kummer D., Milesi S., Mueller D., Joerger M., Aebi S., Amstutz U., Largiader C.R. (2017). Novel Genetic Variants in Carboxylesterase 1 Predict Severe Early-Onset Capecitabine-Related Toxicity. Clin. Pharmacol. Ther..

[31] Frances A., Cordelier P. (2020). The Emerging Role of Cytidine Deaminase in Human Diseases: A New Opportunity for Therapy?. Mol. Ther..

[32] Buhagiar-Labarchède G., Onclercq-Delic R., Vacher S., Berger F., Bièche I., Stoppa-Lyonnet D., Amor-Guéret M. (2022). Cytidine Deaminase Activity Increases in the Blood of Breast Cancer Patients. Sci. Rep..

[33] Ligasová A., Horejšová M., Brumarová R., Friedecký D., Koberna K. (2025). Cytidine and DCMP Deaminases—Current Methods of Activity Analysis. Int. J. Mol. Sci..

[34] Cohen R., Preta L.H., Joste V., Curis E., Huillard O., Jouinot A., Narjoz C., Thomas-Schoemann A., Bellesoeur A., Tiako Meyo M. (2019). Determinants of the Interindividual Variability in Serum Cytidine Deaminase Activity of Patients with Solid Tumours. Br. J. Clin. Pharmacol..

[35] Martín M., Martínez N., Ramos M., Calvo L., Lluch A., Zamora P., Muñoz M., Carrasco E., Caballero R., García-Sáenz J.Á. (2015). Standard Versus Continuous Administration of Capecitabine in Metastatic Breast Cancer (GEICAM/2009-05): A Randomized, Noninferiority Phase II Trial With a Pharmacogenetic Analysis. Oncologist.

[36] Loganayagam A., Arenas Hernandez M., Corrigan A., Fairbanks L., Lewis C.M., Harper P., Maisey N., Ross P., Sanderson J.D., Marinaki A.M. (2013). Pharmacogenetic Variants in the DPYD, TYMS, CDA and MTHFR Genes Are Clinically Significant Predictors of Fluoropyrimidine Toxicity. Br. J. Cancer.

[37] Ciccolini J., Serdjebi C., Peters G.J., Giovannetti E. (2016). Pharmacokinetics and Pharmacogenetics of Gemcitabine as a Mainstay in Adult and Pediatric Oncology: An EORTC-PAMM Perspective. Cancer Chemother. Pharmacol..

[38] Sugiyama E., Lee S.-J., Lee S.S., Kim W.-Y., Kim S.-R., Tohkin M., Ryuichi Hasegawa H.O., Awamoto M.K., Amatani N.K., Awada J.S. (2009). Ethnic Differences of Two Non-Synonymous Single Nucleotide Polymorphisms in CDA Gene. Drug Metab. Pharmacokinet..

