# Supplementary material for: Single-Nucleotide Polymorphisms in Capecitabine Bioactivation Genes and Their Contribution to Breast Cancer Therapy
Source: Pharmaceutics. 2026 May 22;18(6):633. doi: 10.3390/pharmaceutics18060633 (PMC13306124; doi:10.3390/pharmaceutics18060633)
Supplement: Supplementary file 1 [file pharmaceutics-18-00633-s001.zip › pharmaceutics-4274908-supplementary.pdf]

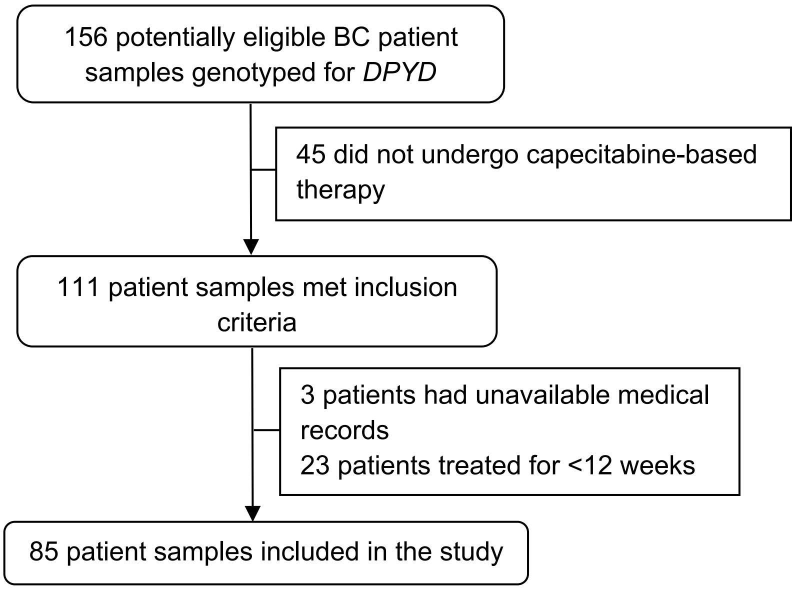

Figure S1. Patient flow diagram

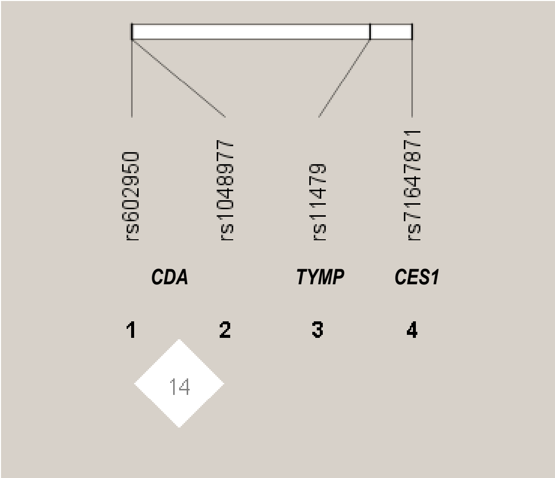

Figure S2. Linkage disequilibrium of selected SNPs. Color scheme: white: D’ < 1, LOD < 2.

Table S1. Minor allele frequency of the selected SNPs.

Chr	SNP	Gene	Major allele	Minor allele	MAF
**1**	*rs602950*	*CDA*	T	C	0.350
**1**	*rs1048977*	*CDA*	C	T	0.280
**16**	*rs71647871*	*CES1*	G	A	0.012
**22**	*rs11479*	*TYMP*	G	A	0.061

Chr: chromosome. SNP: single nucleotide polymorphism. MAF: minor allele frequency.

Table S2. Hardy-Weinberg Equilibrium Analysis of the selected SNPs.

Chr	SNP	Major allele	Minor allele	Genotype counts	Expected heterozygosity	Observed heterozygosity	*p*-value
**1**	*rs602950*	T	C	15/35/35	0.4118	0.4723	0.253
**1**	*rs1048977*	C	T	6/38/41	0.4471	0.4152	0.605
**16**	*rs71647871*	G	A	0/3/82	0.03529	0.03467	1.000
**22**	*rs11479*	G	A	0/16/69	0.1882	0.1705	1.000

Chr: chromosome. SNP: single nucleotide polymorphism.

Table S3. Association of sociodemographic and clinical characteristics with PFS.

Characteristic		Progression-free survival								
		n	Ev.	MST (m)	95% CI	p-value*	Ref. cat	Bivariate Cox model		
								HR	95% CI	p-value
**Family history of cancer**	Yes	59	42	9.47	8.10-13.9	0.600	No	-	-	-
	No	26	17	8.63	6.07-NA					
**Family history of BC**	Yes	32	24	9.17	8.03-19.4	0.600	No	-	-	-
	No	53	35	8.73	7.73-15.0					
**Smoking status**	Smoker	14	10	14.30	1.30-2.01	0.400	Former smoker	-	-	-
	Non-smoker	60	41	37.53	0.32-0.94					
	Former smoker	11	8	7.17	0.10-0.12					
**Alcohol consumption**	Drinker	NA	NA	NA	NA-NA	0.500	Former drinker	-	-	-
	Non-drinker	84	58	58.50	0.0043-0.52					
	Former drinker	1	1	0.50	0.51-0.52					
**Molecular subtype**	Luminal A	15	12	9.17	7.80-NA	0.600	Basal	-	-	-
	Luminal B	35	24	8.27	6.87-26.1					
	HER2+	2	1	12.03	12.03-NA					
	Basal	24	14	12.63	6.47-NA					
**Histopathology**	DCIS	4	3	7.67	4.90-NA	0.300	IDC	-	-	-
	IDC	59	41	9.47	8.03-13.9					
	ILC	12	10	10.80	4.57-NA					
	IC	5	4	5.00	4.13-NA					
	MC	1	0	NA	NA-NA					
**Treatment setting**	Adjuvant	13	2	NA	9.73-NA	**0.020**	Adjuvant	4.41	1.07-18.17	**0.009**
	Non-adjuvant	68	53	8.63	7.80-12					
**Treatment line**	1º	13	2	NA	9.73-NA	**0.050**	Línea 1	1.00<br>3.54<br>4.82	-<br>0.76-16.39<br>1.17-19.93	**0.020**
	2º	14	10	9.47	6.23-NA					
	≥3º	58	47	8.27	7.67-12.6					
**Capecitabine dose reduction**	Yes	39	26	11.30	8.03-16.1	0.300	No	-	-	-
	No	46	32	8.63	7.37-14.1					
**Age at diagnosis (years)**		85	60	-	-	0.600	-	-	-	-
**Daily dose (mg)**		85	60	-	-	0.100	-	-	-	-

95% CI: 95% Confidence interval; BC: breast cancer; DCIS: ductal carcinoma in situ; Ev.: events. HR: hazard ratio. IC: inflammatory carcinoma; IDC: invasive ductal carcinoma; ILC: invasive lobular carcinoma; m: months; MC: mixed mucinous carcinoma; MST: median survival time; n: number of patients; NA: not available. Ref. cat.: reference category; SNP: single nucleotide polymorphism.
* Log-rank p-value.

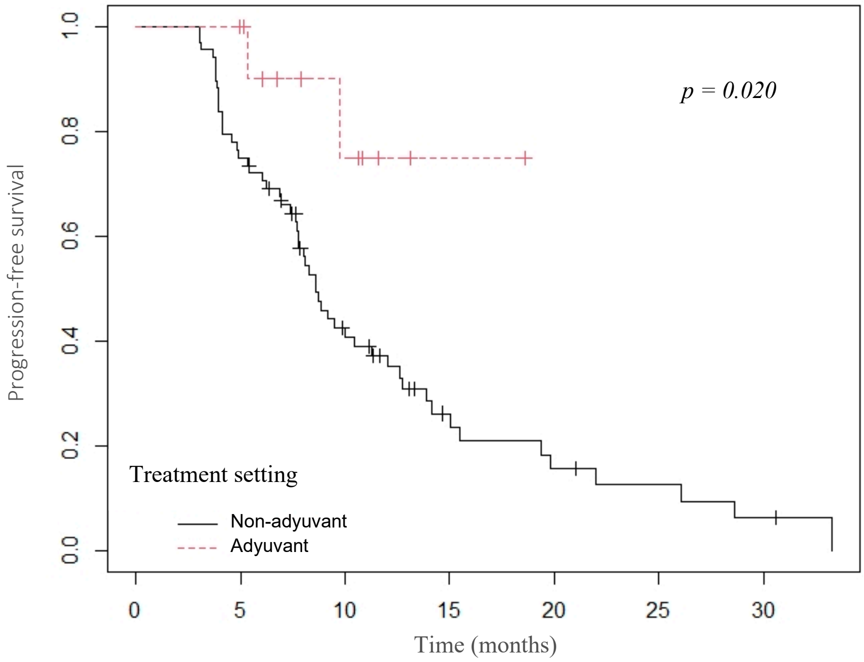

Figure S3. Kaplan-Meier survival curves for PFS according to capecitabine treatment setting (adjuvant vs. non-adjuvant).

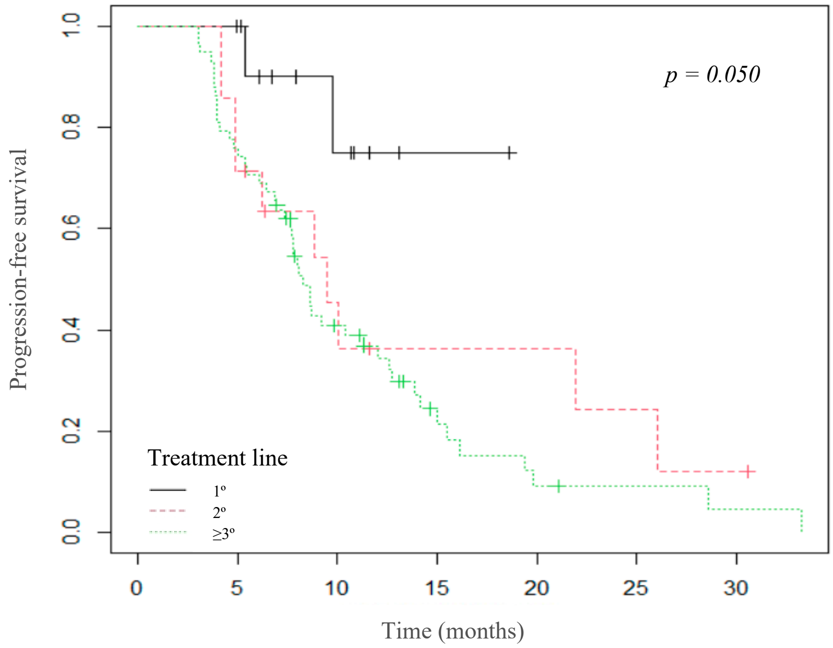

Figure S4. Kaplan-Meier survival curves for PFS according to capecitabine treatment line (first-line vs. ≥third-line).

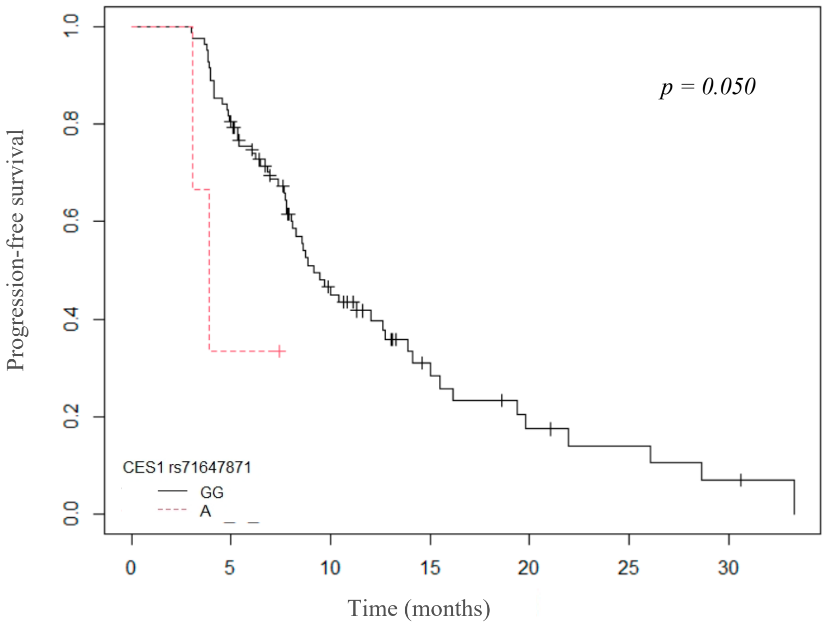

Figure S5. Kaplan-Meier curves for PFS according to the A allele of the *CES1 rs71647871* SNP (GG vs. A).

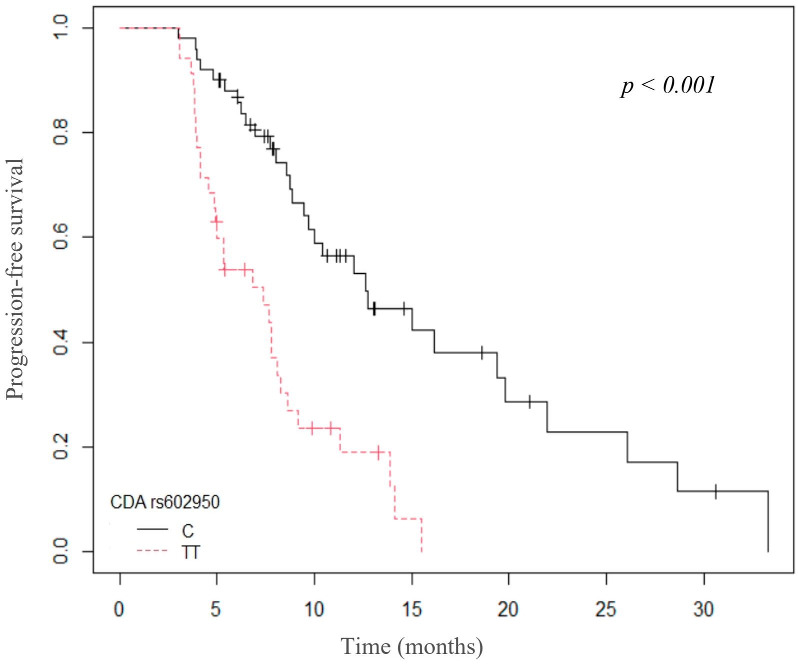

Figure S6. Kaplan-Meier curves for PFS according to the C allele of the *CDA rs602950* SNP (TT vs. C).
